# Supplementary material for: Cell culture-based karyotyping of orectolobiform sharks for chromosome-scale genome analysis
Source: Commun Biol. 2020 Nov 6;3:652. doi: 10.1038/s42003-020-01373-7 (PMC7648076; doi:10.1038/s42003-020-01373-7)
Supplement: Supplementary file 1 — Supplementary Information [file 42003_2020_1373_MOESM1_ESM.pdf]

Supplementary Figures

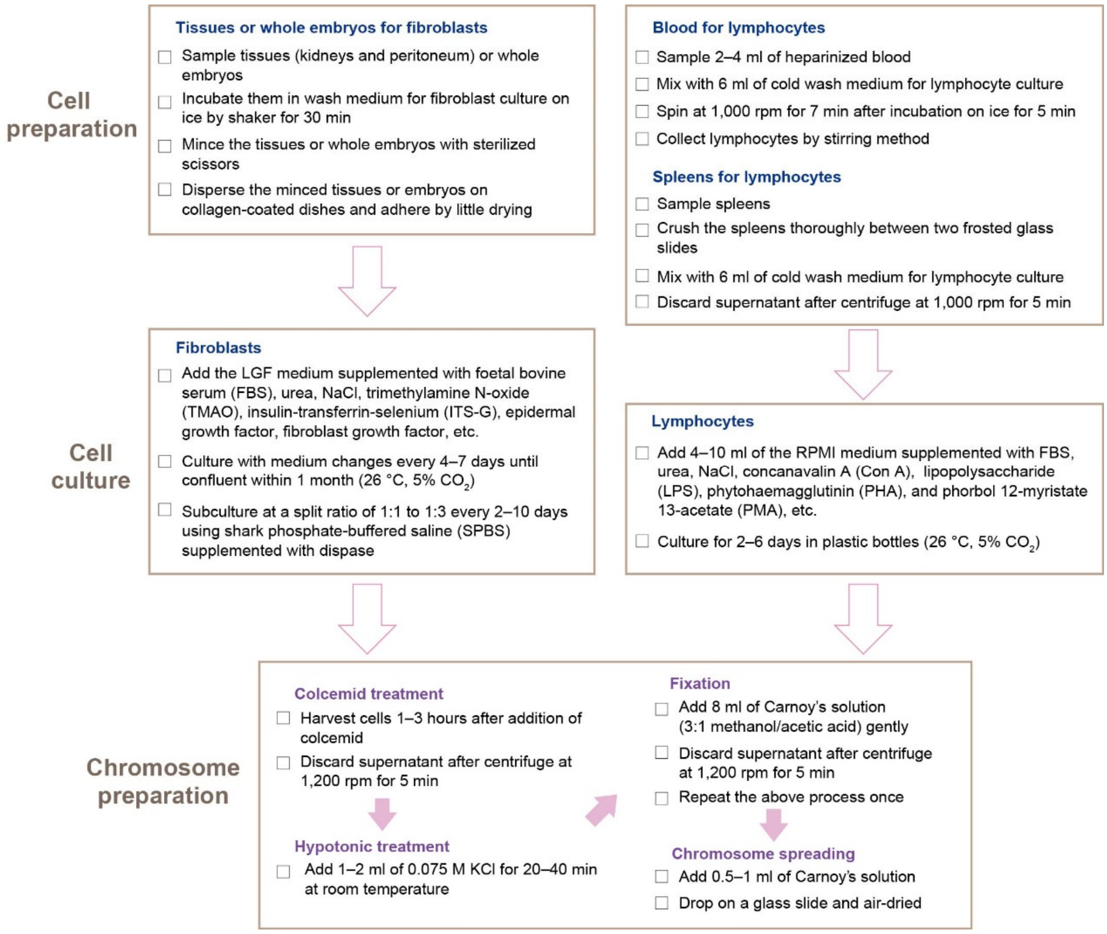

Supplementary Fig. 1. Our cell culture protocol for shark chromosome preparation. See Methods, Results and Supplementary Data 2 for the detail of medium formulations.

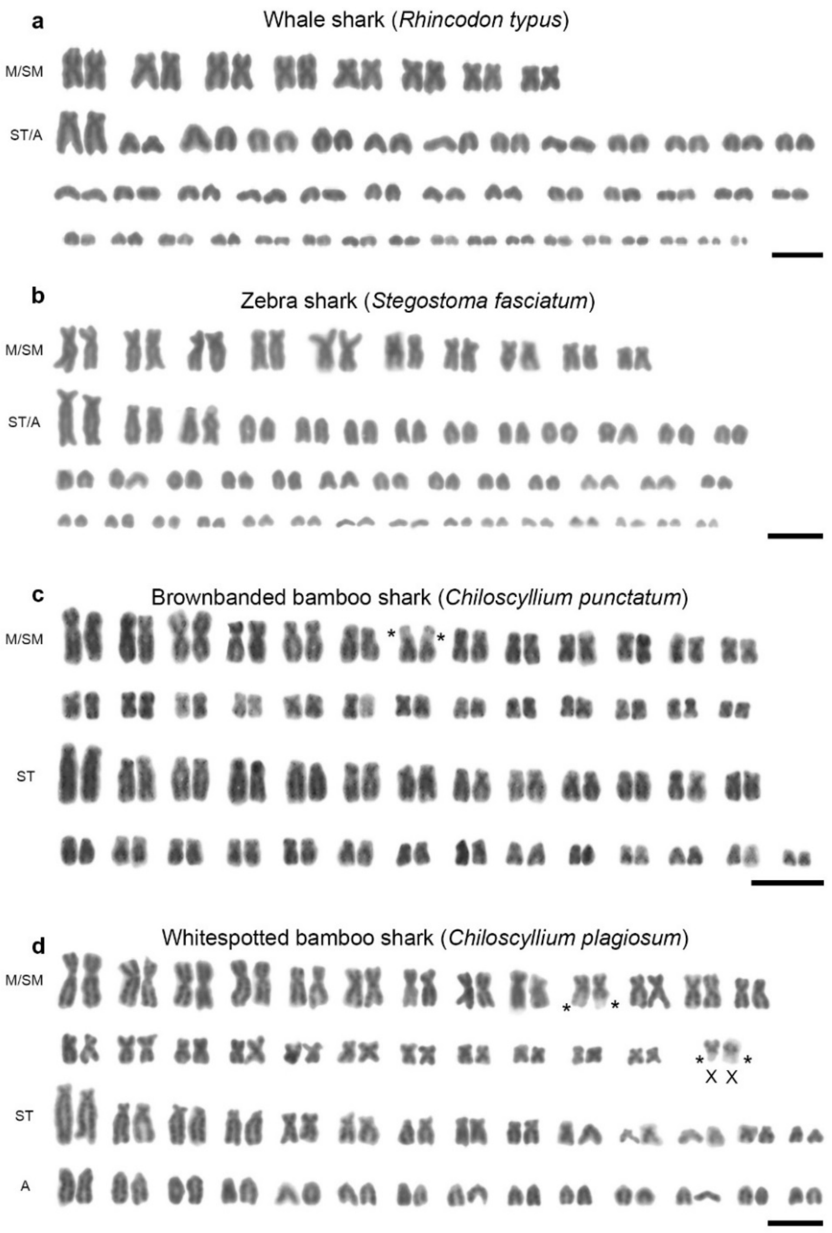

Supplementary Fig. 2. Giemsa-stained karyotypes of female individuals. a Karyotype of a female of the whale shark *Rhincodon typus* (2n = 102). **b** Karyotype of a female of the zebra shark *Stegostoma fasciatum* (2n = 102). **c** Karyotype of a female of the brownbanded bamboo shark *Chiloscyllium punctatum* (2n = 106). **d** Karyotype of a female of the whitespotted bamboo shark *C. plagiosum* (2n = 106). Asterisks indicate the positions of secondary constrictions. M, metacentric chromosomes; SM, submetacentric chromosomes; ST, subtelocentric chromosomes; A, acrocentric chromosomes. Scale bars show 10 µm. See Fig. 3 for male karyotypes and Supplementary Fig. 3 for metaphase spreads.

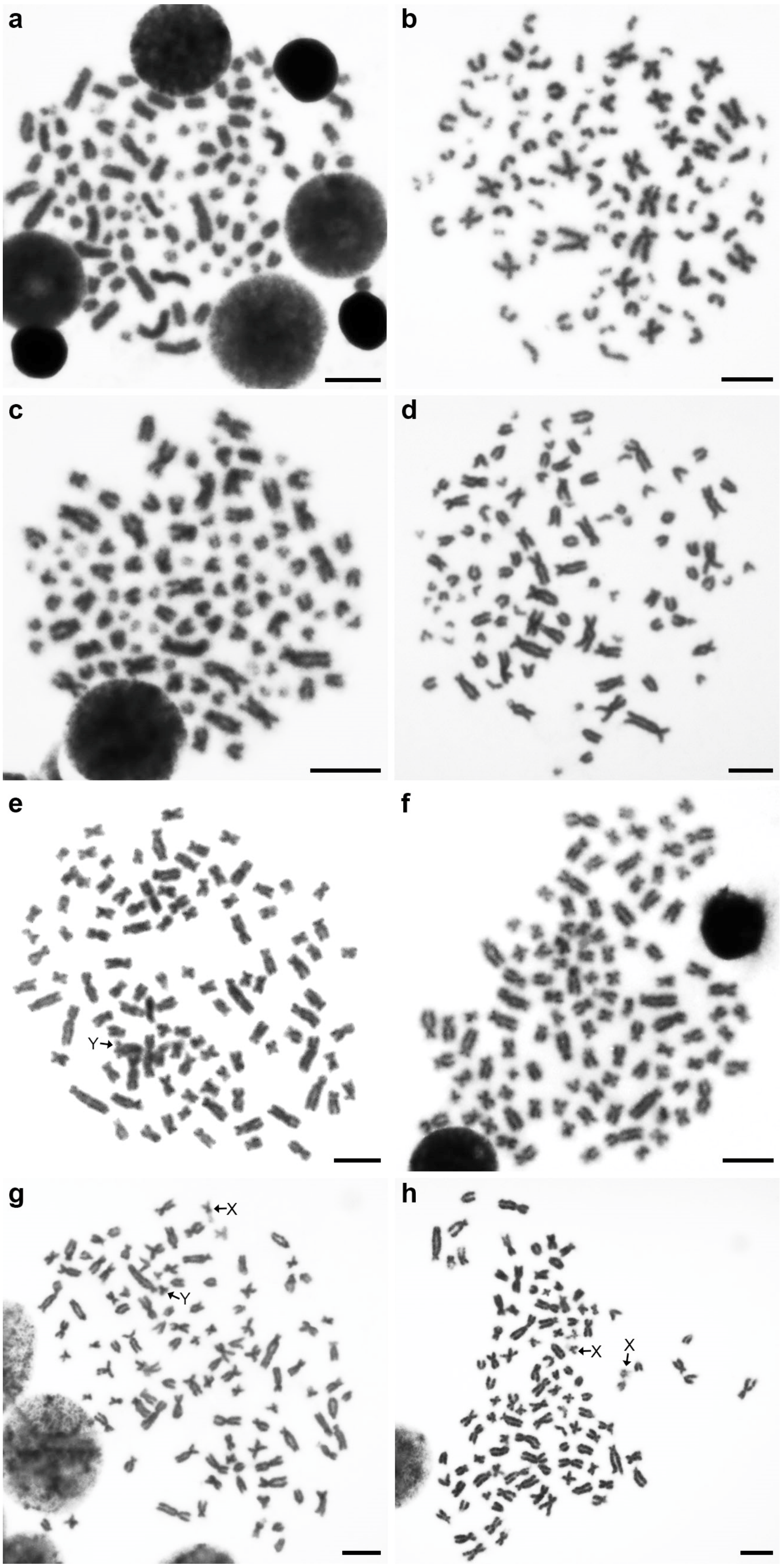

a
b
c
d
e
Y
f
g
X
Y
h
X
X

Supplementary Fig. 3. Giemsa-stained chromosome metaphase spreads. Metaphase spreads of males (**a, c, e, g**) and females (**b, d, f, h**) of the whale shark *Rhincodon typus* (2n = 102) (**a, b**), zebra shark *Stegostoma fasciatum* (2n = 102) (**c, d**), brownbanded bamboo shark *Chiloscyllium punctatum* (2n = 106) (**e, f**), and whitespotted bamboo shark *C. plagiosum* (2n = 106) (**g, h**). The ordered karyotypes from these metaphase spreads are presented in Fig. 3 (for males) and Supplementary Fig. 2 (for females). Arrows indicate putative sex chromosomes (**e, g, h**). Scale bars show 10 μm.

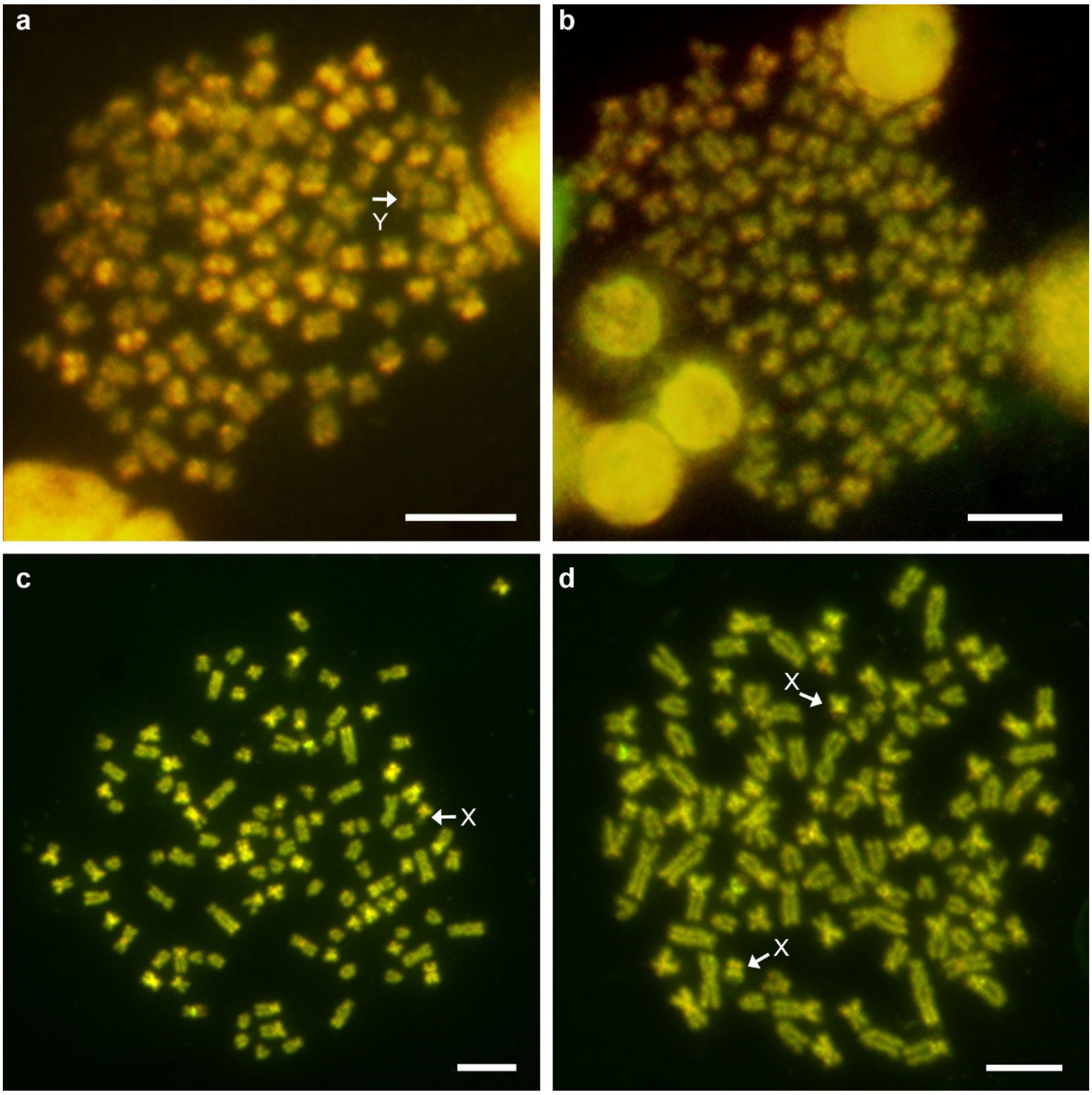

Supplementary Fig. 4. Comparative genomic hybridization. The FITC-labelled female genomic DNA (green) and Cy3-labelled male genomic DNA (red) on metaphase chromosome spreads are shown for a male (**a**) and a female (**b**) of the brownbanded bamboo shark as well as a male (**c**) and a female (**d**) of the whitespotted bamboo shark. Arrows indicate putative sex chromosomes. Scale bars show 10 µm.

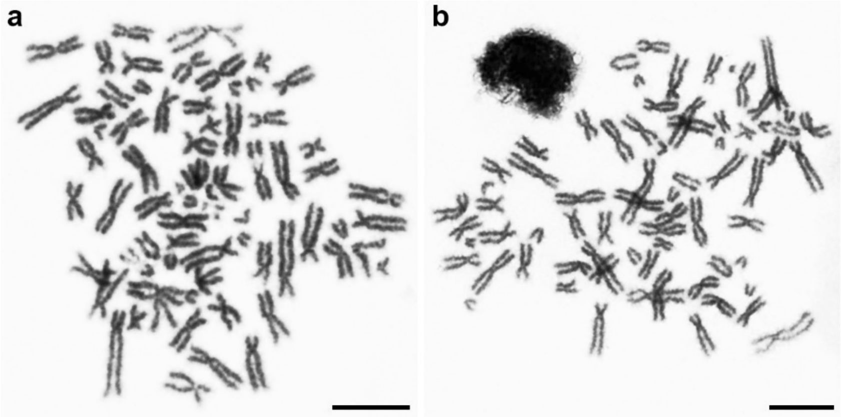

Supplementary Fig. 5. Giemsa-stained chromosome metaphase spreads of two shark species in the order Carcharhiniformes. We performed cell culture from tissues of two shark species in Carcharhiniformes, the banded houndshark *Triakis scyllium* and cloudy catshark *Scyliorhinus torazame* and karyotyping using the cultured cells. A 45 cm-long juvenile of the banded houndshark and an adult of the cloudy catshark were purchased from a commercial marine organism supplier in Mie Prefecture, Japan. After anesthetization with ice cooling, we used kidneys isolated from the banded houndshark for fibroblast culture and a spleen from the cloudy catshark for lymphocyte culture. The cultured cells of the banded houndshark and cloudy catshark were incubated at 26 °C and 20 °C in a humidified atmosphere of 5% CO_2, respectively. The other experimental conditions including medium formulations for cell culture and the method of chromosome preparation are described in the Methods of the main text. Metaphase spreads were obtained from the cultured cells from the banded houndshark (**a**) and cloudy catshark (**b**).These results confirmed their previously reported karyotypes[1,2]. Scale bars show 10 μm.

References in Supplementary Fig. 5

1. Asahida, T., Ida, H. & Inoue, T. Karyotypes and cellular DNA contents of two sharks in the family Scyliorhinidae. *Jpn. J. Ichthyol.* **35**, 215–219 (1988).
2. Asahida, T. & Ida, H. Karyological notes on four sharks in the order Carcharhiniformes. *Jpn. J. Ichthyol.* **36**, 275–280 (1989).

References in Supplementary Data 1 and 2

1. Nygren, A. & Jahnke, M. Microchromosomes in primitive fishes. *Swed. J. Agric. Res.* **2**, 229–238 (1972).
2. Ohno, S. *et al.* Microchromosomes in holocephalian, chondrostean and holostean fishes. *Chromosoma* **26**, 35–40 (1969).
3. Schwartz, F. J. & Maddock, M. B. Comparisons of karyotypes and cellular DNA contents within and between major lines of elasmobranch in *Indo-Pacific Fish Biology* (ed. Uyeno, T., Arai, R., Tuniuchi, T., &Matsuura, K.) 148–157 (Ichthyological Society Japan, 1986).
4. Schwartz, F. J. & Maddock, M. B. Cytogenetics of the elasmobranchs: genome evolution and phylogenetic implications. *Mar. Freshwater Res.* **53**, 491–502 (2002).
5. Asahida, T., Ida, H. & Hayashizaki, K. Karyotypes and cellular DNA contents of some sharks in the order Carcharhiniformes. *Jpn. J. Ichthyol.* **42**, 21–26 (1995).
6. Maddock, M. B. & Schwartz, F. J. Elasmobranch cytogenetics: methods and sex chromosomes. *Bull. Mar. Sci.* **58**, 147–155 (1996).
7. Yabu, H. & Ishii, K. Chromosomes of the great blue shark *Prionace glauce* (Linnaeus). *Bull. Jpn. Soc. Sci. Fish.* **50**, 7–10 (1984).
8. Rocco, L. Sex-related genomic sequences in cartilaginous fish: an overview. *Cytogenet. Genome Res.* **141**, 169–176 (2013).
9. Asahida, T., Ida, H. & Inoue, T. Karyotypes and cellular DNA contents of two sharks in the family Scyliorhinidae. *Jpn. J. Ichthyol.* **35**, 215–219 (1988).
10. Matthey, R. La formule chromosomiale du Selacien *Scyliorhinus catula*. *C. R. Seanc. Soc. Biol.* **126**, 388–389 (1937).
11. Stingo, V. New developments in vertebrate cytotaxonomy II. the chromosomes of the cartilaginous fishes. *Genetica* **50**, 227–239 (1979).
12. Rocco, L., Morescalchi, M. A., Costagliola, D. & Stingo, V. Karyotype and genome characterization in four cartilaginous fishes. *Gene* **295**, 289–298 (2002).
13. Asahida, T. & Ida, H. Karyological notes on four sharks in the order Carcharhiniformes. *Jpn. J. Ichthyol.* **36**, 275–280 (1989).
14. Ida, H., Asahida, T., Yano, K. & Tanaka, S. Karyotypes of two sharks, *Chlamydoselachus anguineus* and *Heterodontus japonicus*, and their systematic implications in *Indo-Pacific Fish Biology* (ed. Uyeno, T., Arai, R., Tuniuchi, T., &Matsuura, K.) 158–163 (Ichthyological Society Japan, 1986).
15. Stingo, V. & Capriglione, T. DNA and chromosomal evolution in cartilaginous fish in *Indo-Pacific Fish Biology* (ed. Uyeno, T., Arai, R., Tuniuchi, T., &Matsuura, K.) 140–147 (Ichthyological Society Japan, 1986).
16. Nygren, A., Nilsson, B. & Jahnke, M. Cytological studies in Hypotremata and Pleurotremata (pisces). *Hereditas* **67**, 275–282 (1971).

17. Makino, S. The chromosomes of two elasmobranch fishes. *Cytologia* **FujiiJubilaei**, 867–876 (1937).
18. Asahida, T. & Ida, H. Karyotypes of two rays, *Torpedo tokionis* and *Dasyatis matsubarai*, and their systematic relationships. *Jpn. J. Ichthyol.* **37**, 71–75 (1990).
19. Donahue, W. H. A karyotypic study of three species of Rajiformes (Chondrichthyes, Pisces). *Can. J. Genet. Cytol.* **16**, 203–211 (1974).
20. Asahida, T., Ida, H. & Inoue, S. Karyotypes of three rays in the order Myliobatiformes. *Jpn. J. Ichthyol.* **33**, 426–430 (1987).
21. Rocco, L., Costagliola, D., Fiorillo, M., Tinti, F. & Stingo, V. Molecular and chromosomal analysis of ribosomal cistrons in two cartilaginous fish, *Taeniura lymma* and *Raja montagui* (Chondrichthyes, Batoidea). *Genetica* **123**, 245–253 (2005).
22. Rocco, L. Molecular markers in cartilaginous fish cytogenetics. in *Fish cytogenetics* (Pisano, E., Ozouf-Costaz, C., Foresti, F. & Kapoor, B. G.) 473–490 (Science Publishers, 2007).
23. Asahida, T., Ida, H., Terashima, H. & Chang, H.-Y. The karyotype and cellular DNA content of a ray, *Mobula japonica*. *Jpn. J. Ichthyol.* **40**, 317–322 (1993).
24. Valentim, F. C. S., Falcão, J. N., Porto, J. I. R. & Feldberg, E. Chromosomes of three freshwater stingrays (Rajiformes Potamotrygonidae) from the Rio Negro basin, Amazon, Brazil. *Genetica* **128**, 33–39 (2006).
25. Valentim, F. C. S., Porto, J. I. R. & Feldberg, E. Chromosomal characterization of Amazonian freshwater stingrays with evidence for new karyomorphs and XX/XY sex chromosomes. *Genet. Mol. Biol.* **42**, 578–593 (2019).
26. Cruz, V. P., Shimabukuro-Dias, C. K., Oliveira, C. & Foresti, F. Karyotype description and evidence of multiple sex chromosome system $X_1X_1X_2X_2/X_1X_2Y$ in *Potamotrygon* aff. *motoro* and *P. falkneri* (Chondrichthyes: Potamotrygonidae) in the upper Paraná River basin, Brazil. *Neotrop. Ichthyol.* **9**, 201–208 (2011).
27. Aichino, D. R. *et al.* Characterization and description of a multiple sex chromosome system in *Potamotrygon motoro* (Chondrichthyes, Myliobatiformes) from the Paraná River, Argentina. *Genet. Mol. Res.* **12**, 2368–2375 (2013).
28. Valentim, F. C. S., Porto, J. I. R., Bertollo, L. A. C., Gross, M. C. & Feldberg, E. XX/XO, a rare sex chromosome system in *Potamotrygon* freshwater stingray from the Amazon Basin, Brazil. *Genetica* **141**, 381–387 (2013).
29. Rocco, L. *et al.* Molecular and karyological aspects of Batoidea (Chondrichthyes, Elasmobranchi) phylogeny. *Gene* **389**, 80–86 (2007).
30. Kikuno, T. & Y. Ojima, 1987. A karyotypic studies of a guitar fish, *Rhinobatos hyinnicephalus* Richardson (Pisces, Rajiformes). *La Kromosomo* II **47–48**: 1538–1544.

31. Asahida, T. & Ida, H. Karyotype and cellular DNA content of a guitarfish, *Rhinobatos schlegelii*. *La Kromosomo* II **79–80**, 2725–2730 (1995).
32. Ida, H., Sato, I. & Miyawaki, N. Karyotypes of two species in the order Torpediniformes. *Jpn. J. Ichthyol.* **32**, 107–111 (1985).
33. Stingo, V., Rocco, L., Odierna, G. & Bellitti, M. NOR and heterochromatin analysis in two cartilaginous fishes by C-, Ag- and RE (restriction endonuclease)-banding. *Cytogenet. Cell Genet.* **71**, 228–234 (1995).
34. Rocco, L. Molecular and Chromosomal Markers for Evolutionary Considerations in Torpediniformes (Chondrichthyes, Batoidea). *ISRN Genetics* **2013**, 1–10 (2013).
35. Garner, W. D. Elasmobranch tissue culture: in vitro growth of brain explants from a shark (*Rhizoprionodon*) and dogfish (*Squalus*). *Tissue Cell* **20**, 759–761 (1988).
36. Hartmann, J. X., Bissoon, L. M. & Poyer, J. C. Routine establishment of primary elasmobranch cell cultures. *In Vitro Cell. Dev. Biol.* **28A**, 77–79 (1992).
37. Poyer, J. C. & Hartmann, J. X. Establishment of a cell line from brain tissue of the silky shark, *Carcharhinus falciformis*. *In Vitro Cell. Dev. Biol.* **28A**, 682–684 (1992).
38. Parton, A. *et al.* Cell and molecular biology of SAE, a cell line from the spiny dogfish shark, *Squalus acanthias*. *Comp. Biochem. Physiol. C Toxicol. Pharmacol.* **145**, 111–119 (2007).
39. Grogan, E. D. & Lund, R. A culture system for the maintenance and proliferation of shark and sting ray immunocytes. *J. Fish Biol.* **36**, 633–642 (1990).
40. McKinney, E. C. Proliferation of shark leukocytes. *In Vitro Cell. Dev. Biol.* **28A**, 303–305 (1992).
41. Maddock, M. B. & Schwartz, F. J. Elasmobranch cytogenetics: methods and sex chromosomes. *Bull. Mar. Sci.* **58**, 147–155 (1996).
42. Walsh, C. J. & Luer, C. A. Comparative phagocytic and pinocytic activities of leucocytes from peripheral blood and lymphomyeloid tissues of the nurse shark (*Ginglymostoma cirratum* Bonaterre) and the clearnose skate (*Raja eglanteria* Bosc). *Fish Shellfish Immunol.* **8**, 197–215 (1998).
43. Walsh, C. J. *et al.* Nitric oxide production by nurse shark (*Ginglymostoma cirratum*) and clearnose skate (*Raja eglanteria*) peripheral blood leucocytes. *Fish Shellfish Immunol.* **20**, 40–46 (2006).
44. Helmrich, A. & Barnes, D. Zebrafish embryonal cell culture. *Methods Cell Biol.* **59**, 29–37 (1999).
45. Uno, Y. *et al.* Divergence of repetitive DNA sequences in the heterochromatin of medaka fishes: Molecular cytogenetic characterization of constitutive heterochromatin in two medaka species: *Oryzias hubbsi* and *O. celebensis*

(Adrianichthyidae, Beloniformes). *Cytogenet. Genome Res.* **141**, 212–226 (2013).
46. Fujiwara, A. *et al.* Improved fish lymphocyte culture for chromosome preparation. *Genetica* **111**, 77–89 (2001).
