# Supplementary material for: Cell culture-based karyotyping of orectolobiform sharks for chromosome-scale genome analysis
Source: Commun Biol. 2020 Nov 6;3:652. doi: 10.1038/s42003-020-01373-7 (PMC7648076; doi:10.1038/s42003-020-01373-7)
Supplement: Supplementary file 3 — Description of Additional Supplementary Files [file 42003_2020_1373_MOESM3_ESM.pdf]

Description of Additional Supplementary Files

Supplementary Data 1: Published chondrichthyan karyotypes.

Supplementary Data 2: Published culture medium formulations of chondrichthyan cells.

Supplementary Data 3: The counts of metaphase spreads in each chromosome number of the four shark species in this study.
