# Supplementary material for: Cell culture-based karyotyping of orectolobiform sharks for chromosome-scale genome analysis
Source: Commun Biol. 2020 Nov 6;3:652. doi: 10.1038/s42003-020-01373-7 (PMC7648076; doi:10.1038/s42003-020-01373-7)
Supplement: Supplementary file 7 — Reporting Summary [file 42003_2020_1373_MOESM7_ESM.pdf]

nature research

Corresponding author(s): Yoshinobu Uno

Last updated by author(s): Sep 18, 2020

Reporting Summary

Nature Research wishes to improve the reproducibility of the work that we publish. This form provides structure for consistency and transparency in reporting. For further information on Nature Research policies, see our Editorial Policies and the Editorial Policy Checklist.

Statistics

For all statistical analyses, confirm that the following items are present in the figure legend, table legend, main text, or Methods section.

n/a	Confirmed	
☐	☒	The exact sample size (*n*) for each experimental group/condition, given as a discrete number and unit of measurement
☐	☒	A statement on whether measurements were taken from distinct samples or whether the same sample was measured repeatedly
☒	☐	The statistical test(s) used AND whether they are one- or two-sided<br>*Only common tests should be described solely by name; describe more complex techniques in the Methods section.*
☒	☐	A description of all covariates tested
☒	☐	A description of any assumptions or corrections, such as tests of normality and adjustment for multiple comparisons
☒	☐	A full description of the statistical parameters including central tendency (e.g. means) or other basic estimates (e.g. regression coefficient) AND variation (e.g. standard deviation) or associated estimates of uncertainty (e.g. confidence intervals)
☒	☐	For null hypothesis testing, the test statistic (e.g. *F*, *t*, *r*) with confidence intervals, effect sizes, degrees of freedom and *P* value noted<br>*Give P values as exact values whenever suitable.*
☒	☐	For Bayesian analysis, information on the choice of priors and Markov chain Monte Carlo settings
☒	☐	For hierarchical and complex designs, identification of the appropriate level for tests and full reporting of outcomes
☒	☐	Estimates of effect sizes (e.g. Cohen's *d*, Pearson's *r*), indicating how they were calculated

Our web collection on statistics for biologists contains articles on many of the points above.

Software and code

Policy information about availability of computer code

Data collection	*Provide a description of all commercial, open source and custom code used to collect the data in this study, specifying the version used OR state that no software was used.*
Data analysis	MEGA X

For manuscripts utilizing custom algorithms or software that are central to the research but not yet described in published literature, software must be made available to editors and reviewers. We strongly encourage code deposition in a community repository (e.g. GitHub). See the Nature Research guidelines for submitting code & software for further information.

Data

Policy information about availability of data

All manuscripts must include a data availability statement. This statement should provide the following information, where applicable:

- Accession codes, unique identifiers, or web links for publicly available datasets
- A list of figures that have associated raw data
- A description of any restrictions on data availability

Partial DNA fragments of the mitochondrial cytochrome oxidase subunit 1 (COI) genes isolated from the brownbanded bamboo shark and whitespotted bamboo shark are deposited in the DDBJ under the accession number LC537758 and LC537759, respectively.

Field-specific reporting

Please select the one below that is the best fit for your research. If you are not sure, read the appropriate sections before making your selection.

- [x] Life sciences
- [ ] Behavioural & social sciences
- [ ] Ecological, evolutionary & environmental sciences

For a reference copy of the document with all sections, see nature.com/documents/nr-reporting-summary-flat.pdf

Life sciences study design

All studies must disclose on these points even when the disclosure is negative.

Sample size	For identification of the karyotypes and sex chromosomes, we sampled as many shark individuals as possible (at least three). But this was limited by the supply of animals, and we intended to reduce the sacrifice of animals.
Data exclusions	We did not exclude data from the manuscript.
Replication	For nucleotide sequences of COX1 genes determined for species identification of the bamboo shark species, the DNA sequencing was performed three times per sample (technical replication). Karyoyping, chromosome mapping by FISH (Fluorescence in situ hybridization), and CGH were examined for at least 25 chromosome metaphase spreads from both sexes for each of the species. These analyses were also performed three times per sample.
Randomization	Karyotyping, chromosome mapping by FISH, and CGH for identification of the karyotypes and sex chromosomes were prepared with uniform protocols and by the same person.
Blinding	The investigator was blinded when karyotyping, chromosome mapping by FISH, and CGH were being prepared.

Reporting for specific materials, systems and methods

We require information from authors about some types of materials, experimental systems and methods used in many studies. Here, indicate whether each material, system or method listed is relevant to your study. If you are not sure if a list item applies to your research, read the appropriate section before selecting a response.

Materials & experimental systems

n/a	Involved in the study	
[ ]	[x]	Antibodies
[x]	[ ]	Eukaryotic cell lines
[x]	[ ]	Palaeontology and archaeology
[ ]	[x]	Animals and other organisms
[x]	[ ]	Human research participants
[x]	[ ]	Clinical data
[x]	[ ]	Dual use research of concern

Methods

n/a	Involved in the study	
[x]	[ ]	ChIP-seq
[x]	[ ]	Flow cytometry
[x]	[ ]	MRI-based neuroimaging

Antibodies

Antibodies used	rhodamine-conjugated anti-digoxigenin Fab fragments (Roche Diagnostics, #11207750910)
Validation	Specificity of rhodamine-conjugated anti-digoxigenin Fab fragments to DIG-labeled DNA fragments hybridized to shark metaphase chromosomes was validated with FISH (Fluorescence in situ hybridization).

Animals and other organisms

Policy information about studies involving animals; ARRIVE guidelines recommended for reporting animal research

Laboratory animals	The study did not involve laboratory animals.
Wild animals	We used animals kept for exhibition in the public aquariums or eggs laid by them (Rhincodon typus in Okinawa Churaumi Aquarium and Osaka Aquarium Kaiyukan, Stegostoma fasciatum in Okinawa Churaumi Aquarium, Chiloscyllium punctatum in Osaka Aquarium Kaiyukan, and Chiloscyllium plagiosum in Suma Aqualife Park in Kobe). We also used a juvenile individual of Chiloscyllium plagiosum, which was purchased from a commercial marine organism supplier in Izunokuni city, Shizuoka Prefecture, Japan, and a juvenile individual of Triakis scyllium and an adult individual of Scyliorhinus torazame from a commercial marine organism supplier in Mie Prefecture, Japan. We did not capture wild animals solely for this study. All individuals of Rhincodon typus, all individuals of Stegostoma fasciatum, and all adult individuals of Chiloscyllium punctatum from which blood was sampled are kept in aquarium tanks. All embryos of Chiloscyllium punctatum, all embryos and a juvenile individual of Chiloscyllium plagiosum, a juvenile individual of Triakis scyllium, and an adult individual of Scyliorhinus torazame were killed after anesthetization by rapid cooling to conduct

tissue dissection for cell culture and/or DNA extraction. The details (age and sex) of the used animals are as follows:

Whale shark, Rhincodon typus:
blood cells of a 4.20 m-long juvenile male, an 8.68 m-long juvenile male, a 6.05 m-long juvenile female, and an 8.04 m-long juvenile female for cell culture.

Zebra shark, Stegostoma fasciatum:
blood cells of three male adults and three female adults for cell culture.

Brownbanded bamboo shark, Chiloscyllium punctatum:
Eight stage 32-34 embryos for cell culture and DNA extraction and blood cells of three male adults and three female adults for cell culture.

Whitespotted bamboo shark, Chiloscyllium plagiosum
Five stage 32-34 embryos for cell culture and DNA extraction and blood cells of a 45 cm-long male juvenile for cell culture and DNA extraction.

Banded houndshark, Triakis scyllium
A 45 cm-long juvenile (unknown sex) for cell culture.

Cloudy catshark, Scyliorhinus torazame
An adult (unknown sex) for cell culture.

Field-collected samples	The study did not involve wild samples collected from the field.
Ethics oversight	Sampling at the aquaria was conducted by veterinary staff in accordance with the Husbandry Guidelines approved by the Ethics and Welfare Committee of Japanese Association of Zoos and Aquariums. All other experiments were conducted in accordance with the institutional guideline Regulations for the Animal Experiments and approved by the Institutional Animal Care and Use Committee of RIKEN Kobe Branch.

Note that full information on the approval of the study protocol must also be provided in the manuscript.
